# The Shape of Corneal Deformation Alters Air Puff–Induced Loading

**DOI:** 10.3389/fbioe.2022.848060

**Published:** 2022-03-30

**Authors:** Atieh Yousefi, Cynthia J. Roberts, Matthew A. Reilly

**Affiliations:** ^1^ Department of Ophthalmology and Visual Sciences, The Ohio State University, Columbus, OH, United States; ^2^ Department of Biomedical Engineering, The Ohio State University, Columbus, OH, United States

**Keywords:** biomechanics, cornea, air puff load, deformation, noncontact tonometry

## Abstract

**Purpose:** To determine the dynamic modification of the load exerted on the eye during air-puff testing by accounting for the deformation of the cornea.

**Methods:** The effect of corneal load alteration with surface shape (*CLASS*) was characterized as an additional component of the load produced during the concave phase where the fluid outflow tangential to the corneal surface creates backward pressure. Concave phase duration (*t*_*CD*_), maximum *CLASS* value (*CLASS*_*max*_), and the area under *CLASS*-time curve (*CLASS*_*int*_) are calculated for 26 keratoconic (KCN), 102 normal (NRL), and 29 ocular hypertensive (OHT) subjects. Tukey’s HSD tests were performed to compare the three subject groups. A p-value less than 0.05 was considered statistically significant.

**Results:** Accounting for *CLASS* increased the load by 34.6% ± 7.7% at maximum concavity; these differences were greater in KCN subjects (*p* < 0.0001) and lower in OHT subjects (*p* = 0.0028) than in NRL subjects. *t*_*CD*_ and *CLASS*_*int*_ were significantly longer and larger, respectively, for KCN subjects than those in the NRL and OHT groups (*p* < 0.0001).

**Conclusion:** Load characterization is an essential step in assessing the cornea’s biomechanical response to air-puff–induced deformation. The dynamic changes in the corneal surface shape significantly alter the load experienced by the corneal apex. This implies a subject-specific loading dynamic even if the air puff itself is identical. This is important when comparing the same eye after a surgical procedure or topical medication that alters corneal properties. Stiffer corneas are least sensitive to a change in load, while more compliant corneas show higher sensitivity.

## Introduction

Corneal biomechanics is an essential tool in corneal disease diagnosis and in providing timely disease management and treatment (Piñero et al., 2010; Terai et al., 2012; Vinciguerra et al., 2016; Kling and Hafezi, 2017). Biomechanical measurements are used in modifying intraocular pressure (IOP) estimation (Liu and Roberts, 2005; Luce, 2006; Elsheikh et al., 2015), assessing the risk of procedures, such as refractive surgery (Liu and Roberts, 2005; Ambrósio et al., 2010; Santhiago et al., 2016), and diagnosing, monitoring, and treating diseases such as keratoconus and glaucoma (Kotecha, 2007; Gorgun et al., 2011).

Characterizing tissue deformation in response to known loading is a common biomechanical diagnostic approach. These techniques can be divided into the two major categories of contact and noncontact loads. For example, atomic force microscopy is a well-established technique for micro-indentation and characterization of living cell stiffness *ex vivo* (Thomas et al., 2013). In other studies, noncontact methods are introduced by oscillating acoustic force by ultrasound transducers (Vappou et al., 2015). An alternative approach more commonly used clinically in ocular applications is deformation using an air puff. This method is also utilized in other areas of study, such as skin stiffness characterization (Boyer et al., 2012).

Specifically for ocular applications, two devices are clinically used to assess corneal biomechanics, both of which do so by using an air puff to deform the cornea and characterize the resulting response. The first device detects bidirectional applanation during deformation and produces parameters such as corneal hysteresis to describe viscoelastic biomechanical response (Luce, 2005). The second device characterizes corneal biomechanical response through Scheimpflug imaging *via* a high-speed camera during the application of a consistent air puff and captures the corneal deformation shape, depth, and timing throughout the imaging period. While both devices provide clinically useful parameters to characterize corneal biomechanics, there still are several confounding parameters that may influence interpretation. Many of these confounding factors, such as IOP and corneal thickness, have been assessed previously (Whitacre et al., 1993; Roberts, 2014). However, the dynamic, nonlinear interaction between the deforming surface shape and the noncontact fluid load has not been assessed previously. Corneal biomechanical parameters are currently interpreted under the assumption of similar load application on different subjects. Detailed knowledge of the applied load is, therefore, essential in interpreting the biomechanical response.

As explained by the Coanda effect, (Benner, 1964) the fluid jet tends to stay attached to the deforming surface, altering the angle of fluid outflow. This back flow, therefore, introduces an additional component of the load on the material surface which may vary between patients or even between tests on the same patient. In this study, we aimed to investigate the underlying interaction between dynamic corneal curvature changes in response to the applied air puff on the load experienced by the cornea. We hypothesized that the load experienced by the corneal apex is altered by the changes in corneal surface shape during the deformation process. In order to investigate this hypothesis, we analytically characterized the effect of changing the geometry on the load amplitude in corneal deformation under an air puff.

## Methods

The effect of changing the geometry on the loading is characterized in CorVis ST (OCULUS Optikgeräte GmbH, Wetzlar, Germany), in a similar method introduced by Tanaka et al. (2011), where they have utilized conservation of mass and conservation of momentum to calibrate the load based on the change in surface curvature and surface deformation on the skin.

### Characterization of Load Under Deformation

**FIGURE 1:**
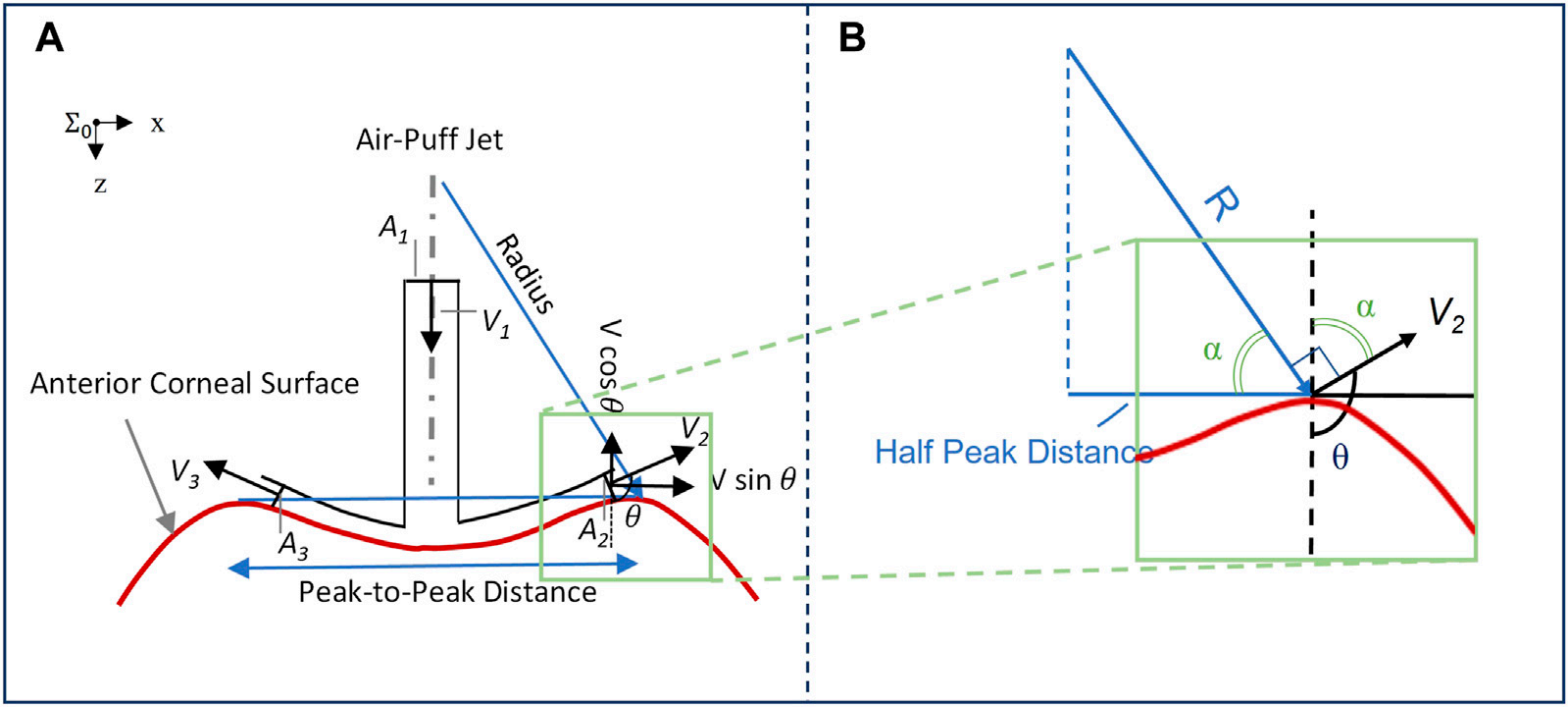
**(A)** Impact of corneal surface shape on the load experienced post applanation, illustrated with a concave cornea. The inlet airflow velocity and cross-sectional area are denoted by V_1_ and A_1_, respectively, while the two-dimensional representation of outlet airflow areas and velocities is denoted by A_2_ and A_3_, and V_2_ and V_3_, respectively; **(B)** Plotting vectors of interest from the same origin to characterize the response angle *θ*.

Tanaka et al. (2011) derived an analytical approximation for load amplification due to the two-dimensional, axisymmetric loading of the skin by an air puff. They found that the load experienced on a concave surface can far exceed that experienced on a flat surface, in this case corresponding to the corneal apex. Specifically, the load in the direction of the air puff z is as follows:

$$F_{z}\left(t\right)={\mathrm{\rho v}}^{2}A\left(1-\cos\left(\theta \right)\right),$$ (1)

where *F*_*z*_ is the load exerted by the air puff and experienced at the corneal apex, *ρ* is the air density, *A* is the cross-sectional area of the device nozzle, and *θ* is the angle between the air puff impacting the cornea normal to the surface and the airflow exiting from the corneal deformation area after interacting with the deformed shape (Figure 1).

### Corneal Load Alteration With Surface Shape Effect Characterization

The impact of the deforming surface on the load experienced by the corneal apex is represented in Figure 1. The values of concave radius and peak-to-peak distance are exported from the CorVis ST research software and used to compute the angle *θ* for each of the 140 video frames captured during the approximately 30-ms duration immediately following the air puff. As shown in Figure 1B, the vector representing the radius is perpendicular to *V*_*2*_ and the line representing half the peak-to-peak distance is perpendicular to the dashed line, implying that the two angles marked with *α* are congruent. We can, therefore, conclude that

$$\cos\alpha =\frac{\mathrm{PD}}{2R}=-\cos\theta ,$$ (2)

where *PD/2* is half the peak-to-peak distance and *R* is the radius of curvature, where the concave values are positive and are exported from CorVis ST and the convex values are negative and are calculated with respect to corneal geometry. All are exported as time-series parameters by CorVis ST Research Software version 1.6r2036 (Research).

Given the coordinate system shown, the positive direction is that of the air puff exiting the device nozzle; therefore, the previously mentioned equation can be rewritten as follows:

$$F_{z}\left(t\right)=\rho v^{2}A\left(1+\frac{\mathrm{PD}}{\mathrm{2R}}\right).$$ (3)

Based on the abovementioned equation, we can characterize corneal load alteration with surface shape (CLASS), which measures the change in the load experienced by the corneal apex due to the dynamic changes in geometry:

$$F_{z}\left(t\right)=\;\rho v^{2}A\left(1+\;CLASS\right),$$ (4)

where *CLASS* is as follows:

$$CLASS=-\cos\theta =\frac{PD}{2R}.$$ (5)

It is to be noted that the CLASS value can, therefore, be bounded between −1 and 1, with zero corresponding to applanation. Thus, *CLASS* indicates a fractional change in the overall air puff loading due to corneal shape: it is negative while the anterior cornea remains convex (i.e., *R > 0*, so the true load is lower than the uncorrected value), and then it becomes positive while the cornea is concave (i.e., *R < 0*, so the true load is higher than the uncorrected value). Time varying parameters include angle *θ* which is a function of PD and R from the Corvis ST and %CLASS. Other CLASS-related parameters, such as CLASSmax, can be extracted from %CLASS time-series and are described in Table 1.

**TABLE 1:** Definitions of CLASS-related parameters.

*CLASS%*	Time series of % change in corneal load alteration with surface shape (CLASS)
*θ*	Angle between airflow impacting the cornea and airflow exiting the area of deformation
*CLASSmax*	Maximum CLASS% value in time-series
*CLASSmin*	Minimum CLASS% value in time-series
*CLASSint*	Integrated Area under the CLASS% curve in the time-series between A1^a^ and A2^b^
*t*_*CD*_	Confinement Duration during the concave phase between A1^a^ and A2^b^

a First applanation.

b Second applanation.

### Patient Data

An analysis was performed on a subset of data acquired from two ongoing studies on corneal biomechanics, each under an approved protocol by The Ohio State University Institutional Review Board (IRB). Informed consent was obtained from all subjects after explanation of the nature and possible consequences of the study. The subset included 157 eyes of 157 subjects, with 26 eyes of 26 subjects diagnosed with keratoconus (KCN), 102 eyes of 102 subjects with normal (NRL) eyes without ocular disease, and 29 eyes of 29 subjects diagnosed with ocular hypertension (OHT). To be eligible, subjects had to be older than 18 years, with a clear cornea in at least one eye. Further group-specific inclusion criteria for the KCN group included a diagnosis of keratoconus having clinical signs such as reduced corneal thickness, steepening, Fleischer’s ring, Vogt’s striae, or scissoring. For the NRL group, subjects with previous or current diagnosis of diabetes mellitus or a history of ocular disease, trauma, or surgery were excluded. For the OHT group, subjects with Goldmann measured IOP greater than 21 mmHg with at least one eye having IOP greater than 24 mmHg were included. The patients were excluded from the study if 1) the subject eye had a nonintact epithelium, 2) the subject was pregnant, less than 12 weeks since post-partum or less than 12 weeks since the completion of breast feeding, 3) the subject eye had nystagmus or any other condition which would prevent a steady gaze at the time of study enrollment, 4) subject had a previous intraocular surgery, except cataracts, 5) subjects with comorbidities that would allow them to be included into more than one of the study cohorts, 6) subjects with systemic conditions that cause defects in collagen (e.g., Marfan’s syndrome, Ehlers-–Danlos syndrome, autoimmune diseases or disorders, etc.), and 7) subjects taking a concomitant medication that could affect result interpretation. All subjects had received a CorVis ST examination along with IOP measurements using dynamic contour tonometry (DCT) (Ziemer Ophthalmic Systems AG; Port, Switzerland).

### CorVis ST Measurements

The previously recorded CorVis ST images are analyzed using research software version 1.6r2036 (Research), and the DCRs are exported. The DCRs utilized in our study include the deformation response at different events of interest, including initial position, first applanation, highest concavity, and second applanation. DCRs describing the changing geometry of the cornea have been shown to be relatively independent of IOP and to correlate with measures of stiffness (Vinciguerra et al., 2016).

IOP, biometry, stiffness, and geometry were also considered. These were, namely, bIOP (biomechanically corrected IOP), central corneal thickness (CCT), stiffness parameters (SPs) at inward applanation (SP-A1), and highest concavity (SP-HC), as well as shape parameters of deformation amplitude (DA) ratio 2 mm and integrated inverse radius. These parameters are calculated as a response to the dynamic load experienced by the cornea over the duration of the examination, as has been shown previously (Joda et al., 2016; Roberts et al., 2017; Roberts and Liu, 2016).

### Statistical Analysis

To evaluate the difference between stiffness metrics in our three subject groups, ANCOVA (analysis of covariates) was performed on stiffness metrics of SP-A1 and SP-HC, while controlling for the effect of age, CCT, and DCT IOP. Furthermore, Tukey’s HSD (honestly significant difference) tests were performed to compare the three subject groups on the maximum *CLASS* value (*CLASS*_*max*_) and concave phase duration (*t*_*CD*_), which is defined as the time between the inward and outward applanation times. To assess the impact of *CLASS*_*max*_ and the area under *CLASS*–time curve, *CLASS*_*int*_, linear regression analyses were performed including these two parameters along with all aforementioned DCRs. Furthermore, regression analyses were performed on *CLASS_max_* and *CLASS*_*int*_ with respect to CCT and bIOP. Next, in order to investigate the influence of stiffness on *CLASS*, a series of regression analyses were performed with *CLASS*_*max*_ and *CLASS*_*int*_ as the independent variable and SP-A1, SP-HC, integrated inverse radius, and DA ratio 2 mm as the dependent variables in all groups. Statistical significance was calculated considering a significance level of 0.05. Statistical analyses were performed in JMP Pro 14.0.0 (SAS Institute Inc, Cary, NC).

## Results

We assessed how this additional component of load, CLASS, impacts the three subject groups and further evaluated *CLASS*-related parameters for the same subject groups; additional analysis was performed to assess the correlation between *CLASS*-related parameters with respect to bIOP, CCT, and current stiffness metrics and shape parameters exported from CorVis ST research software, which are included in the Supplemental Materials.

### Stiffness in Patient Demographics

Mean and standard deviation of each group for age, CCT, and IOP along with the stiffness metrics are shown in Table 2. These parameters were all significantly different between the three groups (*p* < 0.05). ANCOVA results for SP-A1 indicated that when controlling for age, IOP, and CCT, a significant difference exists between all three subject groups (*p*-value < 0.05). On the other hand, ANCOVA for SP-HC when controlling for the effect of age, IOP, and CCT showed significant difference between OHT subjects and the two other groups (*p*-value < 0.0001), while showing no difference between KCN and NRL groups (*p*-value = 0.718).

**TABLE 2:** Subject characteristics for age and IOP, along with stiffness metrics represented by mean ± standard deviation^a^.

	KCN^b^ (*n* = 26)	NRL^c^ (*n* = 102)	OHT^d^ (*n* = 29)
Age	36.73 ± 14.13^†^	45.43 ± 13.74*	60.83 ± 15.12*^,†^
DCT IOP^e^	15.49 ± 3.47^†^	16.95 ± 2.79*	21.29 ± 3.38*^,†^
CCT^f^	496.92 ± 52.85^†^	559.54 ± 31.44*	586.10 ± 41.69*^,†^
SP-A1^g^	73.85 ± 27.25^†^	120.66 ± 18.68	150.42 ± 20.37*
SP-HC^h^	3.77 ± 2.53	7.72 ± 3.72	15.48 ± 5.27*^,†^

a Significant difference between KCN and NRL (*p*-value < 0.05) are shown with *and ^†,^respectively. Stiffness metrics were compared between subject groups while accounting for the effect of age, bIOP, and CCT.

b Keratoconus.

c Normal.

d Ocular hypertensive.

e Dynamic contour tonometry intraocular pressure.

f Central corneal thickness.

g Stiffness parameter at first applanation.

h Stiffness applanation at highest concavity.

### Changes in CLASS-Related Parameters Between Different Disease States

**FIGURE 2:**
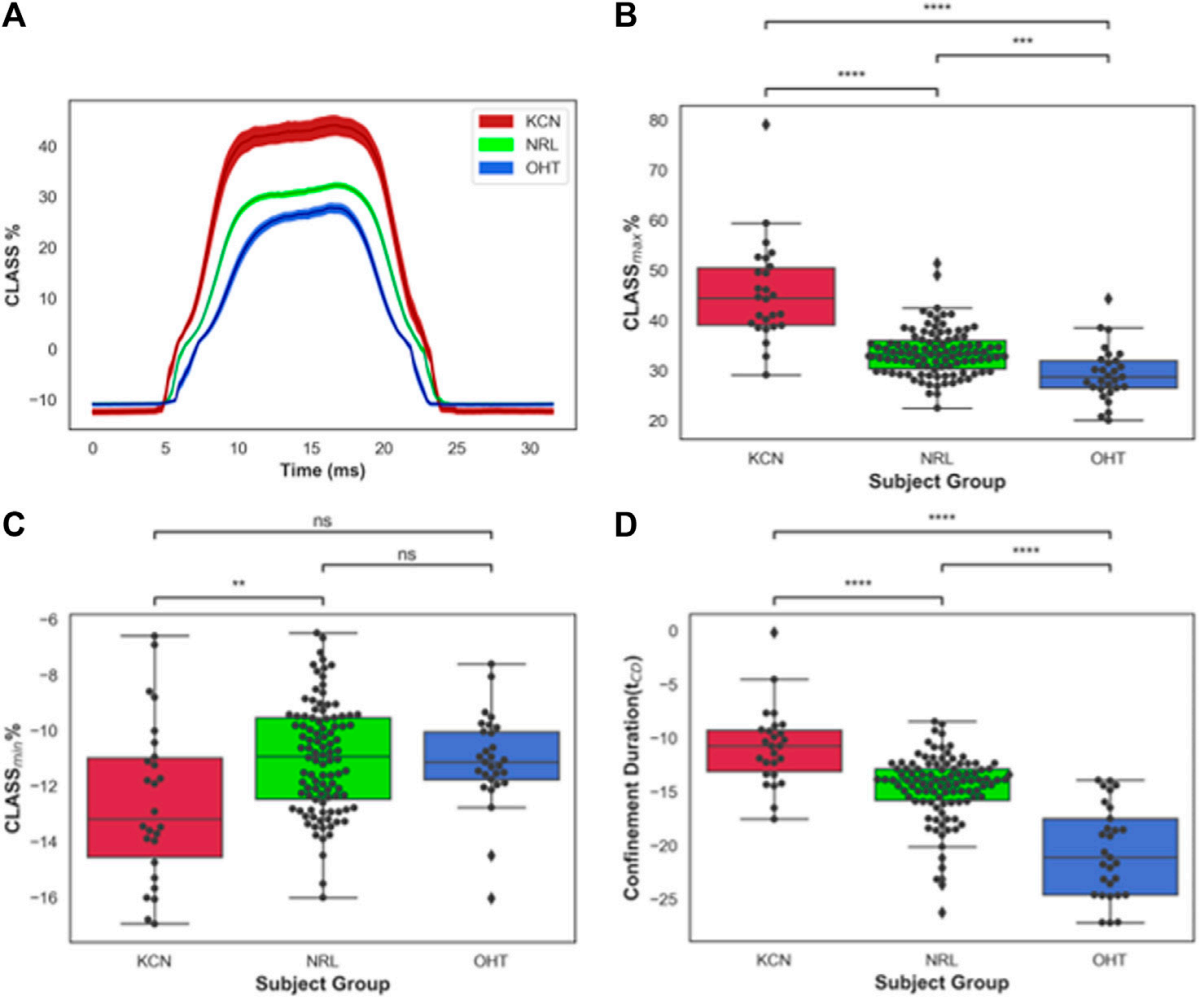
**(A)** *CLASS%* time-series plot shown in mean ± standard error (shaded area) for keratoconus (KCN), normal (NRL), and ocular hypertension (OHT) subjects; **(B)** Distribution of maximum *CLASS* for KCN, NRL, and OHT subjects; **(C)** Distribution of minimum *CLASS* for KCN, NRL, and OHT subjects; **(D)** Distribution of concave phase duration (*t*_*CD*_) for KCN, NRL, and OHT subjects. *P*-value annotation legend: ns: 0.05 < *p*, *: 0.01 < *p* ≤0.05; **: 0.001 < *p* ≤ 0.01; ***: 0.0001 < *p* ≤ 0.001; ****: *p* ≤ 0.0001.

CLASS-related parameters were compared between KCN, NRL, and OHT subjects. Time-series data on CLASS are shown in Figure 2A for all three subject groups. As shown in this figure, the CLASS-adjusted load for KCN subjects indicates that the maximum load experienced by the corneal apex, *CLASS*_*max*_, was on average 45.4% ± 9.98% higher than the flow impinging on a flat rigid surface (*p-value < 0.0001*). *CLASS*_*max*_ is on average 33.3% ± 4.28% and 29.2% ± 5.33% higher than the load on a flat rigid surface for NRL and OHT subject groups, respectively (*p-value < 0.0001*).

Furthermore, *CLASS*_*max*_ is shown in Figure 2B for all subjects. A pairwise comparison of all groups using Tukey’s HSD for *CLASS*_*max*_ indicates significantly different values for each pair, with KCN having significantly higher values than NRL and OHT subjects (*p-value < 0.0001*). OHT subjects had significantly lower *CLASS*_*max*_ than NRL subjects (*p-value = 0.00012*). Figure 2C shows the minimum value, CLASS_*min*_, is dependent primarily on the shape of the unloaded cornea, which was significantly lower in KCN than normal controls.

The concave phase duration, represented with *t*_*CD*_ shown in Figure 2D, was calculated to be 14.92 ± 0.90 ms for KCN subjects, 14.20 ± 0.91 ms for NRL subjects, and 12.54 ± 1.01 ms for the OHT group. Tukey’s HSD tests performed on *t*_*CD*_ showed significantly shorter duration for OHT subjects than that of KCN and NRL subjects (*p-value < 0.0001*), with the KCN subjects having significantly higher *t*_*CD*_ than NRL subjects (*p-value = 0.0015*).

**FIGURE 3:**
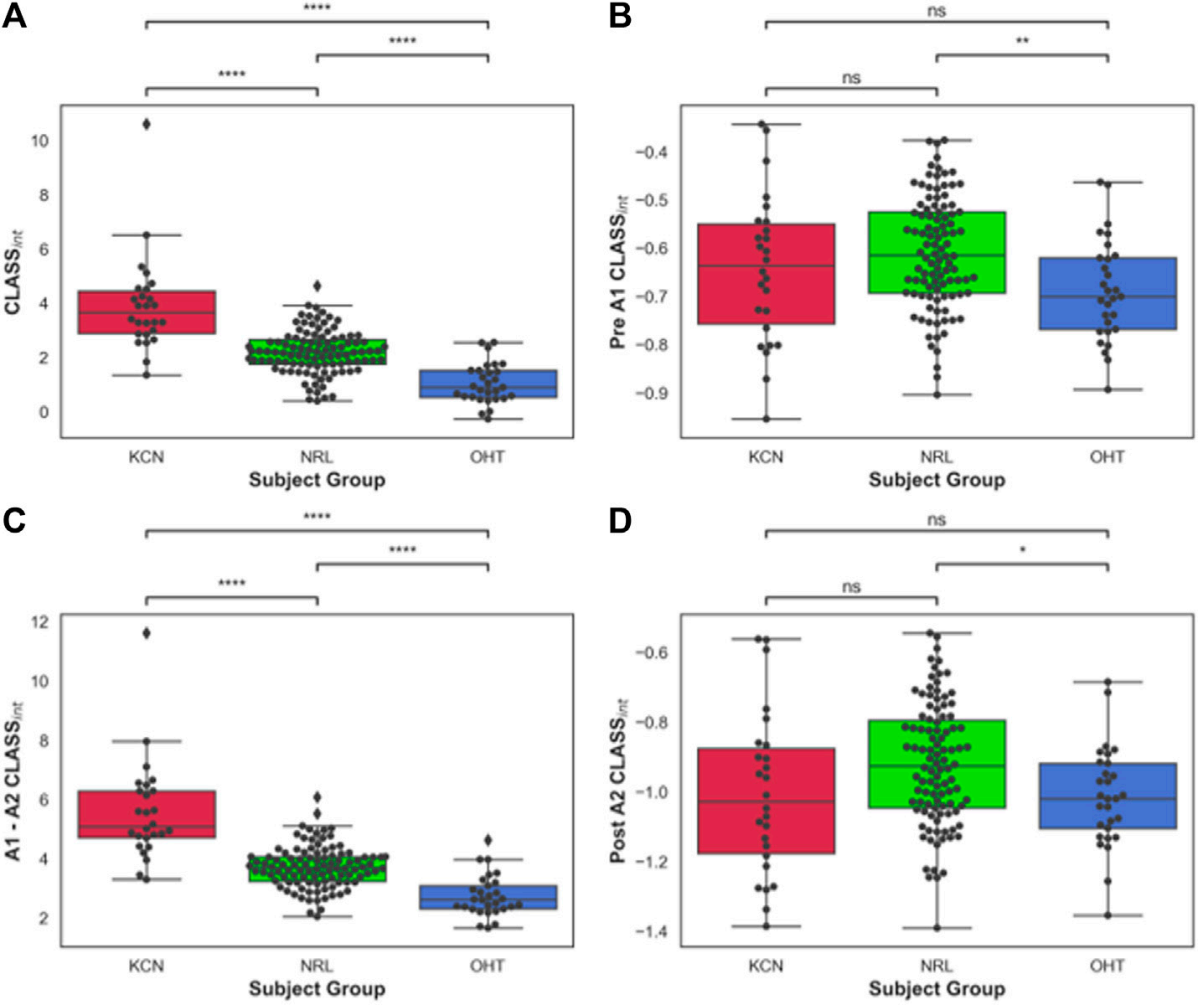
**(A)** Area under *CLASS* curve (*CLASS*_*nt*_) prior to first applanation for keratoconus (KCN), normal (NRL), and ocular hypertension (OHT) subjects; **(B)** *CLASS*_*int*_ between the two applanation points for KCN, NRL, and OHT subjects; **(C)** *CLASS*_*int*_ after second applanation for KCN, NRL, and OHT subjects. **(D)** Total *CLASS*_*int*_ for KCN, NRL, and OHT subjects. *P*-value annotation legend: ns: 0.05 < *p*, *: 0.01 < *p* ≤ 0.05; **: 0.001 < *p* ≤ 0.01; ***: 0.0001 < *p* ≤ 0.001; ****: *p* ≤ 0.0001.

The area under the *CLASS*-time curve was calculated and integrated *CLASS* (*CLASS*_*int*_) was plotted for all subjects (Figure 3A). CLASS_int_ was on average 5.48 ± 1.66 for the KCN group, 3.67 ± 0.7 for NRL subjects, and 2.69 ± 0.68 for OHT subjects. Tukey’s HSD test comparing the difference in *CLASS*_*int*_ for each ocular condition shows significant difference in *CLASS*_*int*_ between all three groups (*p-value < 0.0001*), with KCN having the highest values and OHT having the lowest values, on average.

To better account for the negative *CLASS* values prior to A1 and after A2, *CLASS*_*int*_ was divided into three sections: before A1 (Figure 3B), between A1 and A2 (Figure 3C), and after A2 (Figure 3D). OHT and NRL groups were significantly different from each other in terms of *CLASS*_*int*_ before A1 and after A2, with *p*-values of 0.0036 and 0.026, respectively. *CLASS*_*int*_ for these two regions was not significantly different between KCN and the other two groups. On the other hand, the three groups were significantly different from each other when comparing *CLASS*_*int*_ for the concave duration between A1 and A2 (*p-value* < 0.0001).

### The Effect of IOP on CLASS

**FIGURE 4:**
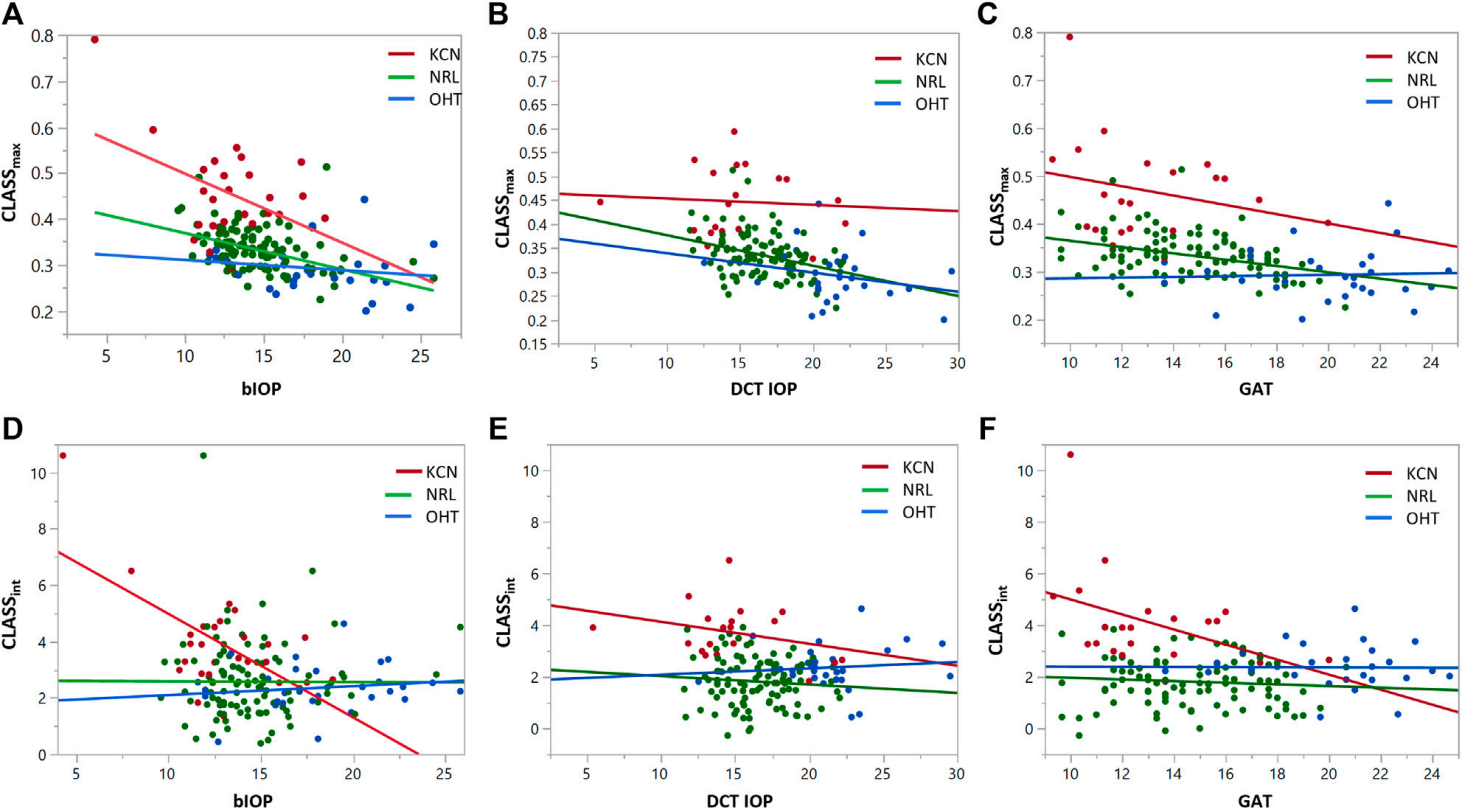
Regression analysis for *CLASS*_*max*_ and *CLASS*_*int*_ with respect to bIOP **(A,D)**, DCT IOP **(B,E)** and GAT **(C and F)**, for keratoconic (KCN), normal (NRL), and ocular hypertensive (OHT) subjects.

Regression analysis of *CLASS*_*max*_ with bIOP is represented by a negative linear relationship for KCN and NRL groups, while showing no significant correlation for OHT (KCN*: R* ^*2*^ *= 0.2029*, *p < 0.0001*, NRL: *R* ^*2*^ *= 0.2415*, *p = 0.209*, OHT: *R* ^*2*^ *= 0.0255*, *p = 0.4081*). Regression analysis of *CLASS*_*max*_ with DCT IOP was represented by a negative linear relationship for all NRL subjects and no correlation for the other two groups (KCN: *R* ^*2*^ *= 0.3866*, *p < 0.0001*, NRL: *R* ^*2*^ *= 0.2270*, *p = 0.0007*, OHT: *R* ^*2*^ *= 0.4342*, *p < 0.0001*). Regression analysis of *CLASS*_*int*_ vs. bIOP showed significant negative relationship for the KCN subject group, while resulting in no correlation for NRL and OHT groups (KCN: *R* ^*2*^ *= 0.3877*, *p = 0.0007*, NRL: *R* ^*2*^ *= 0.0000*, *p = 0.9616*, OHT: *R* ^*2*^ *= 0.0204*, *p = 0.4587*). For DCT IOP, regression analysis showed no correlation for the three groups (KCN: *R* ^*2*^ *= 0.3192*, *p = 0.0026*, NRL: *R* ^*2*^ *= 0.0386*, *p = 0.0488*, OHT: *R* ^*2*^ *= 0.1252*, *p = 0.0597*). The detailed results of each regression analysis are provided in Table 3 and Figure 4.

**TABLE 3:** Regression results for *CLASS*_*max*_ and *CLASS*_*int*_ vs. IOP.

	KCN^a^ (*n* = 26)	NRL^b^ (*n* = 102)	OHT^c^ (*n* = 29)
*CLASS*_*max*_ ^d^			
bIOP^e^	$R^{2}=0.2029,\;P\;<\;.0001$	$R^{2}=0.2415,\;P\;=\text{ 0}.0209$	$R^{2}=0.0255,\;P\;=\text{ 0}.4081$
DCT^f^	$R^{2}=0.3866,\;P\text{ < }.0001$	$R^{2}=0.2270,\;P\;=\text{ 0}.0007$	$R^{2}=0.4342,\;P\;=\text{ 0}.0001$
*CLASS*_*int*_ ^g^			
bIOP	$R^{2}=0.3877,\;P\;=\text{ 0}\text{.}0007$	$R^{2}=0.0000,\;P\;=\text{ 0}\text{.}9616$	$R^{2}=0.0204,\;P\;=\text{ 0}.4587$
DCT	$R^{2}=0.3192,\;P\;=\text{ 0}\text{.}0026$	$R^{2}=0.03865,\;P\;=\text{ 0}.0488$	$R^{2}=0.1252,\;P\;=\text{ 0}.0597$

a Keratoconus.

b Normal.

c Ocular hypertensive.

d Maximum CLASS, index.

e Biomechanically corrected IOP.

f Dynamic contour tonometry intraocular pressure.

g Integrated CLASS, index.

### How is CLASS Correlated With Other DCRs?

**FIGURE 5:**
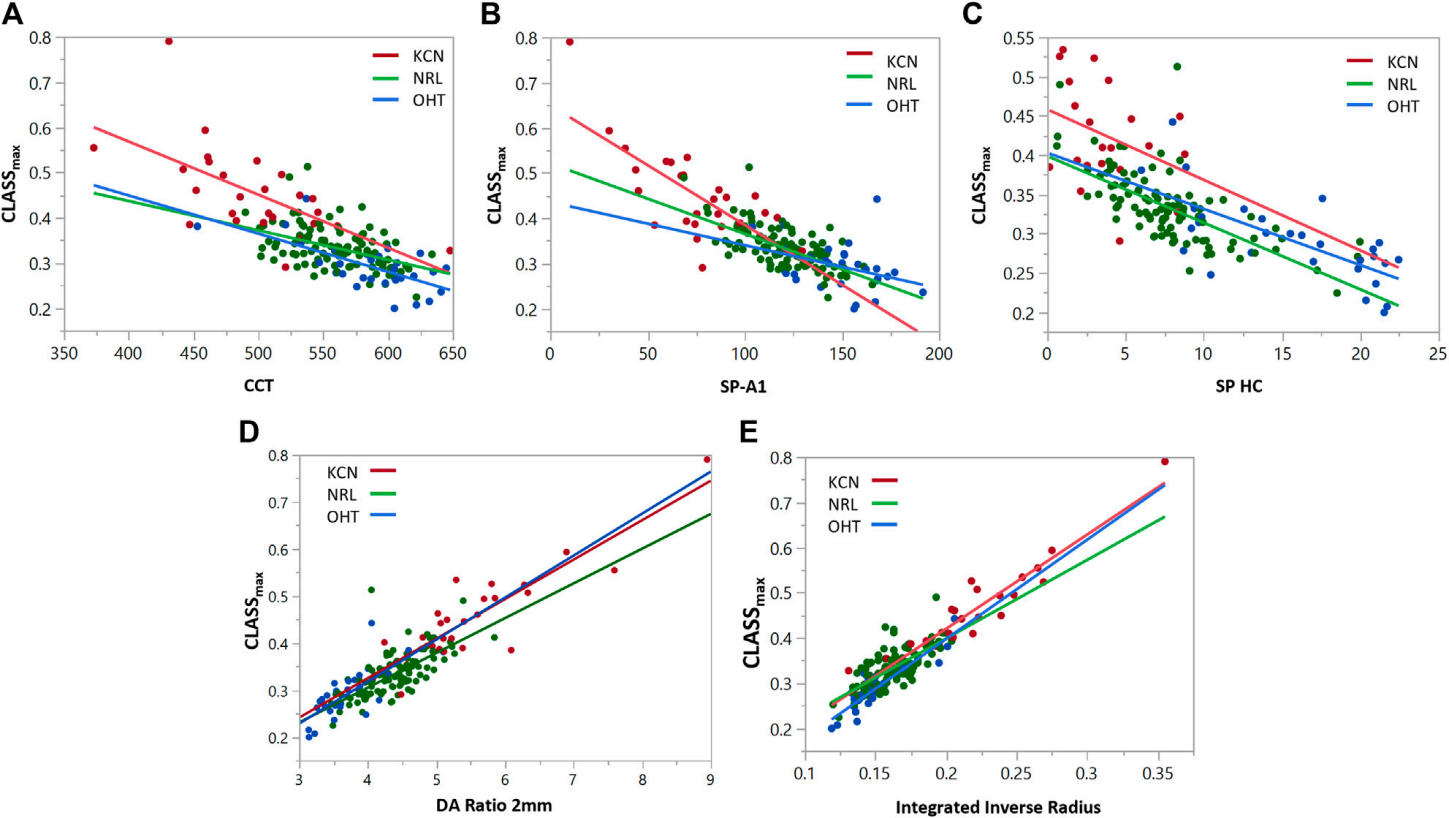
Regression analyses for *CLASS*_*max*_ vs. **(A)** CCT, **(B)** SP-A1, **(C)** SP-HC, **(D)** DA ratio 2 mm, and **(E)** integrated inverse radius for keratoconic (KCN), normal (NRL), and ocular hypertensive (OHT) subjects.

**FIGURE 6:**
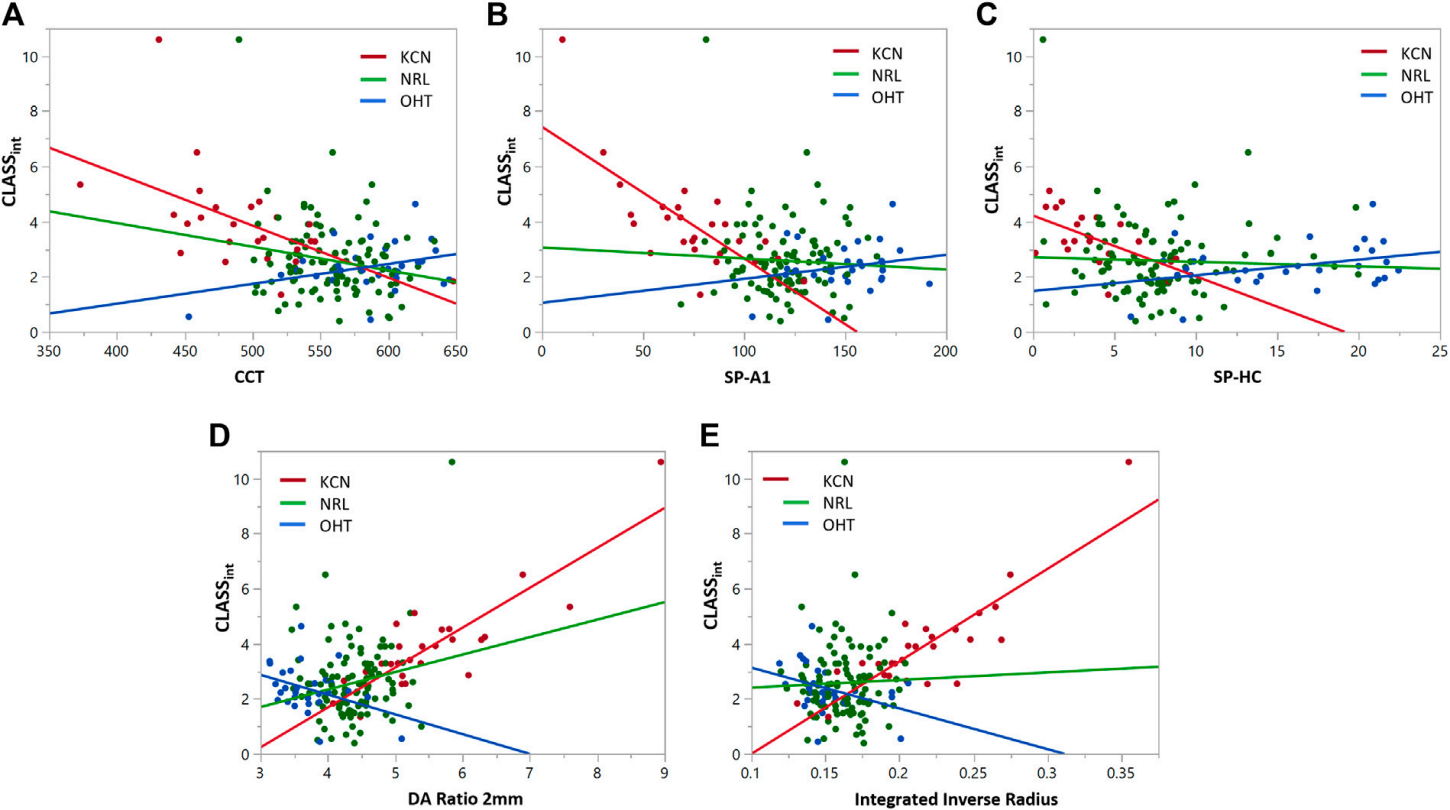
Regression analyses for *CLASS*_*int*_ vs. **(A)** CCT, **(B)** SP-A1, **(C)** SP-HC, **(D)** DA ratio 2 mm, and **(E)** integrated inverse radius for keratoconic (KCN), normal (NRL), and ocular hypertensive (OHT) subjects.

To assess the relationship between the newly introduced *CLASS* parameters and stiffness metrics, regression analyses are performed for *CLASS*_*max*_ in Figure 5 and *CLASS*_*int*_ in Figure 6, with respect to SP-A1, SP-HC, CCT, DA ratio 2 mm, and integrated inverse radius. The details are summarized in Table 4 **.** Regression analysis of *CLASS*_*max*_ with respect to CCT showed significant negative relationships for KCN and NRL groups, as well, while indicating no correlation for the OHT group (KCN: *R* ^*2*^ *= 0.3866*, *p = 0.0026*, NRL: *R* ^*2*^ *= 0.2270*, *p = 0.0488*, OHT: *R* ^*2*^ *= 0.4342*, *p = 0.0597*). For the SP-A1, regression analysis shows a significant negative linear relationship between the two parameters for KCN and NRL subject groups, while the relationship is not significant for the OHT group (KCN: *R* ^*2*^ *= 0.5211*, *p* < 0.0001, NRL: *R* ^*2*^ *= 0.4611*, *p* < 0.0001, OHT: *R* ^*2*^ *= 0.1330*, *p* = 0.0518). Regression analysis for *CLASS* and SP-HC showed a negative linear relationship this time for NRL and OHT subjects and not for KCN (KCN: *R* ^*2*^ *= 0.1212*, *p* = 0.1219, NRL: *R* ^*2*^ *= 0.5426*, *p* < 0.0001, and OHT: *R* ^*2*^ *= 0.5000*, *p* < 0.0001). As shown in Figures 5D,E, linear regression analysis for *CLASS*_*max*_ vs. DA ratio 2 mm (KCN: *R* ^*2*^ *= 0.7812*, *p* < 0.0001, NRL: *R* ^*2*^ *= 0.5754*, *p* < 0.0001, and OHT: *R* ^*2*^ *= 0.5493, p* < 0.0001) and integrated inverse radius (KCN: *R* ^*2*^ *= 0.8982, p* < 0.0001, NRL: *R* ^*2*^ *= 0.5079*, *p* < 0.0001, and OHT: *R* ^*2*^ *= 0.8407, p* < 0.0001) showed a positive linear relationship for all subject groups.

**TABLE 4:** Regression analysis results for *CLASS*_*max*_ and *CLASS*_*int*_ vs. stiffness parameters.

	KCN^a^ (*n* = 26)	NRL^b^ (*n* = 102)	OHT^c^ (*n* = 29)
*CLASS*_*max*_ ^d^			
CCT^e^	$R^{2}=0.3866,P\;\text{<}\;.0001$	$R^{2}=0.2270,\;P\;=\text{ 0}.0007$	$R^{2}=0.4342,\;P\;=\text{ 0}.0001$
SP-A1^f^	$R^{2}=0.5211,P<.0001$	$R^{2}=0.4611,P\;\text{<}\;.0001$	$R^{2}=0.1330,P\;\text{<}\;.0518$
SP-HC^g^	$R^{2}=0.1212,P=0.1219$	$R^{2}=0.5426,P<.0001$	$R^{2}=0.50,P\;\text{<}\;.0001$
DA ratio 2 mm^h^	$R^{2}=0.7812,P\;\text{<}\;.0001$	$R^{2}=0.5754,P\;\text{<}\;.0001$	$R^{2}=0.5493,P\;\text{<}\;.0001$
Integrated inverse radius	$R^{2}=0.8982,P\;\text{< }.0001$	$R^{2}=0.5080,P\;\text{<}\;.0001$	$R^{2}=0.8407,P\;\text{<}\;.0001$
*CLASS*_*int*_ ^i^			
CCT	$R^{2}=0.3192,P\;=\;\text{0}\text{.}0026$	$R^{2}=0.03865,P\;=\;\text{0}.0488$	$R^{2}=0.1252,P\;=\;\text{0}.0597$
SP-A1	$R^{2}=0.5424,P\;\text{<}\;.0001$	$R^{2}=0.0030,P\;=\;\text{0}\text{.}5878$	$R^{2}=0.0436,P\;=\;\text{0}\text{.}2772$
SP-HC	$R^{2}=0.3389,P\;=\;\text{0}\text{.}0056$	$R^{2}=0.0020,P\;=\;\text{0}.6550$	$R^{2}=0.1251,P\;=\;\text{0}\text{.}0598$
DA ratio 2 mm	$R^{2}=0.7451,P\;\text{<}\;.0001$	$R^{2}=0.0416,P\;=\;\text{0}\text{.}0409$	$R^{2}=0.1403,P\;=\;\text{0}\text{.}0453$
Integrated inverse radius	$R^{2}=0.7453,P\;\text{<}\;.0001$	$R^{2}=0.0013,P\;=\;\text{0}\text{.}7254$	$R^{2}=0.1508,P\;=\;\text{0}\text{.}0374$

a Keratoconus.

b Normal.

c Ocular hypertensive.

d Maximum CLASS, index.

e Central corneal thickness.

f Stiffness parameter at first applanation.

g Stiffness applanation at highest concavity.

h Deformation amplitude ratio at 2 mm.

i Integrated CLASS, index.

Similar statistical analyses were repeated to assess the correlation between *CLASS*_*int*_ with the stiffness and shape metrics, shown in Figure 6. Regression analysis between integrated *CLASS* and CCT showed significant negative correlation for KCN and NRL groups, while indicating no correlation for OHT subjects (KCN: *R* ^*2*^ *= 0.3192*, *p* = 0.0026, NRL: *R* ^*2*^ *= 0.03865*, *p* = 0.0488, and OHT: *R* ^*2*^ *= 0.1252*, *p* = 0.0597). Similar analysis on the stiffness metrics of SP-A1 and SP-HC showed significant negative correlation for the KCN subject group (*R* ^*2*^ *= 0.5424 and R* ^*2*^ *= 0.3389*, and *p* < 0.0001 and *p* = 0.0056, respectively), while showing no significant correlation for NRL and OHT groups. Regression analysis between DA ratio 2 mm and *CLASS*_*int*_ showed significant correlation between the two parameters for all three subject groups (KCN: *R* ^*2*^ *= 0.7451*, *p* < 0.0001, NRL: *R* ^*2*^ *= 0.0416*, *p* = 0.0409, and OHT: *R* ^*2*^ *= 0.1403, p* = 0.0453). Furthermore, regression analysis between *CLASS*_*int*_ and integrated inverse radius showed significant correlation for KCN and OHT subject groups, while showing no correlation for NRL subjects (KCN: *R* ^*2*^ *= 0.7453*, *p* < 0.0001, NRL: *R* ^*2*^ *= 0.0013*, *p* = 0.7254, and OHT: *R* ^*2*^ *= 0.1508, p* 0.0374).

## Discussion

Noncontact deformation analysis is an effective clinical approach used to characterize corneal biomechanics in different settings. This study sheds light on an important yet previously unknown confounding effect with noncontact air puff–induced deformation between the fluid-based load magnitude and corneal surface shape. The results presented in this work exhibit significant changes in the load as a response to the corneal surface shape that may alter not only the clinical interpretations but also results from computer simulations of the response to the applied load. To derive the interlinked relationship between corneal surface shape and fluid-based load, we introduced a new nondimensional factor calculated *via* an analytical approach, previously proposed for characterization of skin tissue stiffness with an indentation test using noncontact air puff impingement (Tanaka et al., 2011). The additional component of the load introduced here, *CLASS*, characterizes the %change in the load experienced at the corneal apex based on corneal dynamic shape changes.

Overall, for the 157 subjects in this study, adjusting for the load due to the surface shape resulted in a 34.57% ± 7.7% increase in the maximum load experienced by the corneal apex. The reason behind the modified load is the change in the air puff outflow angle due to the convex corneal shape before and after the two applanation events and the concave corneal shape between the two applanation events, which in turn alters the impact experienced on the apex. This considerable change in the load indicates the significant effect of the nonlinear and dynamic changes in corneal geometry on the load itself. These findings are in agreement with Tanaka et al.‘s analytical approximation to this dynamic relationship between the load and deformation in a noncontact stiffness sensor used to characterize skin stiffness through air puff indentation (Tanaka et al., 2011). Their findings further explained that the significant impact of recalibrating an air puff load based on the dynamic concave deformation of soft tissue can increase up to two-fold in response to tissue deformation.

The relationship between *CLASS*_*max*_ and bIOP for KCN subjects showed that lower bIOP values will experience a larger load with the concave surface shape as explained by CLASS (Figure 4A **)**. This association was less profound with NRL subjects and further showed no correlation for OHT subjects. Such behavior may indicate that bIOP measurement could be influenced by the dynamic changes in the corneal surface shape and the extent of this influence can be captured through *CLASS*_*max*_ and *CLASS*_*int*_. Furthermore, regression analyses exhibited a negative correlation for *CLASS*_*max*_ with the stiffness metrics, indicating that stiffer corneas are associated with lower changes in load, while *CLASS*_*max*_ exhibited a positive correlation with shape DCRs, which attests to the accuracy of *CLASS* by showing larger changes in the load with larger shape changes.

On the contrary, when assessing the correlation of *CLASS*_*int*_ with these parameters, NRL and OHT subjects reflected a different pattern in comparison with using *CLASS*_*max*_. The reason behind this behavior is likely that *CLASS*_*int*_ is more comprehensive in considering the effect of concavity duration, corneal surface shape before first applanation, after second applanation, and the overall dynamic changes in the surface shape with the load application. Consequently, the shorter *t*_*CD*_, smaller DA ratio and smaller integrated inverse radius in stiffer OHT subjects led to lower *CLASS*_*int*_ (shown in Figures 6C,D). In short, *CLASS*_*int*_ in KCN subjects is driven by the corneal behavior between the two applanation points, while for OHT and NRL subjects, this value is driven by areas outside the two applanation points.

Besides the overall impact of *CLASS* in changing the load experienced by the corneal apex, our study revealed that the confounding effect of corneal surface shape on the load may result in misinterpretation of corneal response in disease states. It is known that keratoconic corneas have reduced the number of collagen lamellae and altered the orientation of these lamellae (Daxer and Fratzl, 1997), which leads to focal weakening and a more compliant response. These characteristics were shown to impact *CLASS*-related parameters by increasing *CLASS*_*max*_ due to the more compliant response and increased corneal deflection between the two applanation events. This indicates the critical effect of altered load in disease states since a higher load applied to advanced keratoconic eyes can be misinterpreted as a larger corneal response. Furthermore, this behavior elucidates the importance of *CLASS* in clinical studies considering that the load applied to the corneal apex changes between subjects with different corneal conditions and within subjects before and after a corneal procedure, such as refractive surgery, or the development of corneal disease. While many clinical studies have focused on assessing corneal biomechanics using a dynamic Scheimpflug analyzer, our findings suggest that these analyses may require further evaluations to correct for the effect of *CLASS* and eliminate the confounding role of dynamic surface shape and load in interpreting corneal biomechanical response.

Findings of our work may impact the results of various clinical studies that have utilized uncorrected air puff load values to monitor KCN (Tian et al., 2014; Elham et al., 2017), evaluate corneal crosslinking (Bak-Nielsen et al., 2014; Tian et al., 2014), assess the risk of refractive surgery (Kataria et al., 2019), assess corneal response pre/post refractive surgery (Pedersen et al., 2014; Frings et al., 2015; Sefat et al., 2016; Xu et al., 2017), or compare different methods of refractive surgery (Pedersen et al., 2014; Sefat et al., 2016). Furthermore, the significant change in the air puff load as a response to the deformed corneal surface is neglected in many modeling studies of noncontact corneal deformation (Francis et al., 2019; Jannesari et al., 2019). Although a previous modeling study reported changes in the fluid dynamics characteristics as the cornea deformed, the specifics of these changes were not quantified (Kling et al., 2014). Ignoring this effect may result in misinterpreting the higher load applied to the cornea as a larger corneal response and may in turn generate misleading corneal characterization.

There are limitations to the present study. The current study was limited by a small sample size for OHT and KCN subjects. In addition, the accuracy of CLASS is limited by several factors. First, we utilized the Bernoulli equation in the sub-sonic flow regime (Mach number ∼0.3), slightly beyond the incompressible flow regime, where it more rigorously holds. Second, we availed ourselves of the existing geometric parameters already characterized by the CorVis ST software; deviation from the spherical cap geometry will introduce minor errors into the CLASS correction. Third, the presence of localized heterogeneities in the corneal deformation (e.g., keratoconus) could cause the cornea to deviate from the assumed spherical cap geometry. Furthermore, the deformation information is based on one corneal meridian and does not take into account the asymmetric corneal shape specifically for KCN subjects. Nevertheless, the analysis presented here clearly demonstrates that despite delivering a consistent air puff, the resulting dynamic load experienced by the eye can differ significantly.

This study identifies an additional component of the air puff load, *CLASS*, to indicate how the dynamic change in corneal surface shape can alter the load experienced by the cornea. A major impact of *CLASS* is that while the dynamic Scheimpflug analyzer applies a consistent air puff with each examination, the load experienced by the cornea can be different for each subject, depending on the shape of the corneal deformation, which may ultimately affect the characterization of corneal biomechanics. Stiffer corneas of OHT subjects are associated with a lower change in the load, while more compliant corneas of KCN subjects are associated with larger load alterations. Hence, correcting the load to account for corneal surface geometry is an essential initial step to interpret corneal biomechanical behavior. How to specifically include CLASS in this interpretation remains an area of future study, as well as determining the three-dimensional nature of air puff backflow.

## Data Availability

The raw data supporting the conclusion of this article will be made available by the authors, without undue reservation.
